# Removal of toxic Cr(VI) from aqueous medium with effective magnetic carbon-based nanocomposites

**DOI:** 10.55730/1300-0527.3629

**Published:** 2023-09-30

**Authors:** Ferda CİVAN ÇAVUŞOĞLU, Gülsüm ÖZÇELİK, Şahika Sena BAYAZİT

**Affiliations:** 1 Department of Chemical Engineering, Faculty of Engineering and Architecture, İstanbul Beykent University, İstanbul, Turkiye; 2 Department of Nanotechnology, Nanotechnology and Biotechnology Institute, İstanbul University-Cerrahpaşa, İstanbul, Turkiye

**Keywords:** Adsorption, carbon nanocomposites, graphene nanoplatelet, hexavalent chromium, magnetic adsorbents

## Abstract

Cr(VI), which has toxic effects, is a heavy metal and it must be removed from the environment due to the various damages it causes. In this study, the removal of Cr(VI) pollutants from aqueous solutions with Fe_3_O_4_-based materials using a batch adsorption technique was investigated. Magnetically modified graphene nanoplatelet (GNP)-based nanocomposites were prepared and their structures were characterized by FTIR, XRD, SEM, BET, and TGA techniques. The effects of various physicochemical parameters such as adsorbent dose, contact time, initial Cr(VI) solution concentration, pH, and the presence of coexisting ions (NaCl) on the adsorption process were investigated. Accordingly, the optimum conditions for Cr(VI) removal were determined. Nonlinear Langmuir, Freundlich, and Temkin isotherm models and pseudo-first-order, pseudo-second-order, and Bangham kinetic models were used to investigate the adsorption mechanism. The experimental data relatively fit the second-order kinetic model and the Freundlich isotherm model. The maximum adsorption capacities for pure Fe_3_O_4_ (Fe:GNP 1:0), Fe:GNP (2:1), and Fe:GNP (1:1) nanocomposite materials at 298 K and pH of approximately 5 were obtained as 12.71 mg/g, 27.03 mg/g, and 62.27 mg/g, respectively. This result showed that Cr(VI) removal increased as the amount of GNP in the composite material increased. Generally, the results confirmed that magnetically modified GNP-based adsorbents are functional and promising materials that can be used for the removal of pollutants such as Cr(VI) from aqueous media.

## 1. Introduction

The effects of heavy metals on environmental pollution and the health of living things are increasing day by day and becoming a major problem worldwide. Heavy metals have high density and toxicity at ppb levels [1]. Chromium (Cr), one of the heavy metals, is a transition metal found in water in valences ranging from +6 to −2. The most stable forms are the hexavalent Cr(VI) and trivalent Cr(III) forms, which are interchangeable [2]. Cr(III) is generally found in the inorganic form (Cl^−^, NO_3_^-^, etc.) or in complexes with organic ligands (RCOOH, etc.), while Cr(VI) exists as strong oxidizing agents (CrO_3_, CrO_4_^2−^, and Cr_2_O_7_^2−^) [1,3]. Cr(III) is generally absorbed by the soil and it precipitates in soil, preventing its mobility and accumulation in the environment. Cr(III) has low solubility in water, while Cr(VI) is a carcinogen with high solubility in water and strong oxidation and must be removed from wastewater. Cr(VI) is therefore highly dynamic in terrestrial and aquatic environments and can be found in the natural environment for a longer time. Harmful effects of Cr(VI) ions on the skin, eyes, kidneys, lungs, and gastrointestinal and central nervous systems have been observed in living organisms, including cancer and other disorders [2,4–7]. Cr(VI) is used in catalyst production, pigments and dyes, photography, leather, the ceramic and glass industries, and corrosion control. Chromium is present in water as a result of municipal wastewaters, industrial dewatering, agricultural actions, mining processes, petrochemical materials, and battery production [8]. Due to its high toxicity, it is among the top 20 pollutants in lists of hazardous chemicals. For these reasons, it is important to remove Cr(VI) pollution from wastewaters.

Chemical precipitation, membrane filtration processes, reverse osmosis, electrodialysis, ion exchange, flotation, and photocatalysis are among the methods used for removing Cr(VI) from wastewaters [9–11]. Although these methods are highly effective, many of them cause environmental pollution at a high cost, which limits their practical application [12,13]. Adsorption, which is a traditional method, is accepted as a practical and economical method for Cr(VI) removal from water owing to its flexibility and suitability for most real-world wastewater treatment situations. In addition, the simplicity of design, ease of use, and possibility of the regeneration of adsorbents are advantages of this method [14,15].

In addition to conventional activated carbon, Cr(VI) removal from water has been studied using promising adsorbents including carbon nanotubes [14,16], kaolinite [16–20], biochar [5,21–24], lignin [25,26], diatomite [27–29], lignite [30–33], graphene oxide [34–37], natural zeolites [38–40], and clay [41,42].

Graphene nanoplatelets (GNPs) have the advantage of selective adsorption for organic and inorganic pollutants as a result of electrostatic and hydrogen bond interactions [43]. They have large surface areas, balanced electrical charges, ease of processing, and high environmental stability [44,45]. The use of nanoparticles to remove contaminants from water can make separation difficult or introduce the possibility of particles escaping into the water stream. Magnetic nanocomposites have been suggested as promising materials for water treatment due to properties such as better dispersibility and stability, ease of separation from water, and economic value and environmental friendliness compared to free nanoparticles. They also facilitate the treatment of major volumes of wastewater in a short time without harmful byproducts. Iron oxides (Fe_2_O_3_ and Fe_3_O_4_) are unique and capable magnetic components that form new compounds with graphene. Their effective use in separation processes has increased the interest in magnetic adsorbents [46–48]. Various studies have been carried out with magnetic adsorbents for Cr(VI) removal [49–73].

In this study, magnetically modified GNP-based nanocomposites were prepared as an adsorbent for Cr(VI) uptake from water; thus, they were easily separated from aqueous solutions after adsorption. Adsorbents with different Fe:GNP ratios were prepared and their effects on the adsorption process were examined comparatively. FTIR, SEM, XRD, BET, and TGA analyses were performed to determine the structural and thermal properties of adsorbents. The effects of different factors such as adsorbent dose, contact time, initial Cr(VI) solution concentration, pH, and presence of different ions (NaCl) on the adsorption process were investigated in Cr(VI) removal. To understand the adsorption mechanism, pseudo-first-order (PFO), pseudo-second-order (PSO), and Bangham nonlinear kinetic models and Langmuir, Freundlich, and Temkin isotherm models were investigated by applying them to the experimental data.

## 2. Materials and methods

### 2.1. Chemicals

GNPs (xGnP®-C-750, thickness: 1–20 nm, width: 1–50 μm) were received from XG Sciences (Lansing, MI, USA). Potassium dichromate (K_2_Cr_2_O_7_), iron(II) sulfate heptahydrate (Fe(SO_4_)·7H_2_O), iron(III) chloride hexahydrate (FeCl_3_·6H_2_O), HCl (36.5%), NaOH (≥99%), NaCl, and ammonium hydroxide (NH_4_OH) solution (25%) were purchased from Merck Co. (Darmstadt, Germany).

### 2.2. Preparation of magnetic nanocomposites

Three different magnetic nanocomposite materials were obtained using the coprecipitation method [48,74]. Magnetic nanocomposites (pure Fe_3_O_4_ (Fe:GNP 1:0), Fe:GNP (2:1), and Fe:GNP (1:1)) were prepared such that the ratio of [Fe(II) + Fe(III)]:GNP was 1:0, 2:1, and 1:1, respectively. Fe(II) and Fe(III) ions were obtained from FeCl_3_·6H_2_O and Fe(SO_4_)·7H_2_O hydrate compounds. Certain proportions of GNP were also added and the mixtures were kept in an ultrasonic bath. NH_4_OH solution (8 M) was slowly added until the pH of the mixture reached approximately 11–12. The precipitates that formed were washed 5–6 times with distilled water. Finally, the obtained product was passed through ethanol and dried in a vacuum oven at 60 °C.

### 2.3. Characterization of nanocomposites

FTIR, SEM, XRD, and TGA analyses were performed to characterize the structural and thermal properties of the synthesized magnetic nanocomposites. FTIR analyses were carried out by turning the samples into pellets with the KBr method using a Bruker Alpha device (Bruker, Billerica, MA, USA). The morphology of the nanocomposites was observed with a Vega 3 scanning electron microscope (SEM; Tescan, Warrendale, PA, USA) at 10,000× magnification. The surface of the samples was sputter-coated with a gold-palladium layer for SEM visualization. The specific surface areas of the nanocomposites were determined by N_2_ adsorption–desorption isotherms using the BET method on a NOVAtouch device (Quantachrome, Boynton Beach, FL, USA). XRD analyses were performed with a Rigaku D/Max-2200 diffractometer (Rigaku Corp., Tokyo, Japan) and the intensities of data from the nanocomposites were measured in the range of 20° to 60° at the 2θ angle with a scanning rate of 2°/min. TGA analyses were performed using the STA-7200 simultaneous thermogravimetric analyzer (Hitachi, Tokyo, Japan) with heating under nitrogen gas from room temperature to 1000 °C at a rate of 10 °C/min.

### 2.4. Adsorption studies

There are many physicochemical parameters that affect the adsorption process. To determine the optimum adsorption conditions, the effects of different factors such as adsorbent dose, contact time, pH, presence of different ions, and Cr(VI) solution initial concentration on the adsorption system were investigated. In adsorption studies performed as a batch process, the volume of the 25 ppm Cr(VI) solution was kept constant at 10 mL, prepared from K_2_Cr_2_O_7_. Adsorption experiments were carried out with 100 mL Erlenmeyer flasks in an orbital shaker with agitation of 120 rpm. After the experiments, the magnetic nanocomposites were easily separated from the Cr(VI) solution with the help of magnets. Concentrations of the remaining Cr(VI) solution were measured at 351 nm with a V-730 UV-Vis spectrophotometer (Jasco, Tokyo, Japan).

Adsorption capacity is a significant parameter for adsorption processes and is calculated with the formula shown in Eq. (1), where Co (mg/L) is the initial concentration of Cr(VI), Ce (mg/L) is the equilibrium concentration, m (g) is the amount of adsorbent, and V (L) is the solution volume.


(1) $$\text{qe}=\frac{(\text{Co}-\text{Ce})\text{.V}}{\text{m}}$$

## 3. Results and discussion

### 3.1. Characterization studies

**Figure 1:**
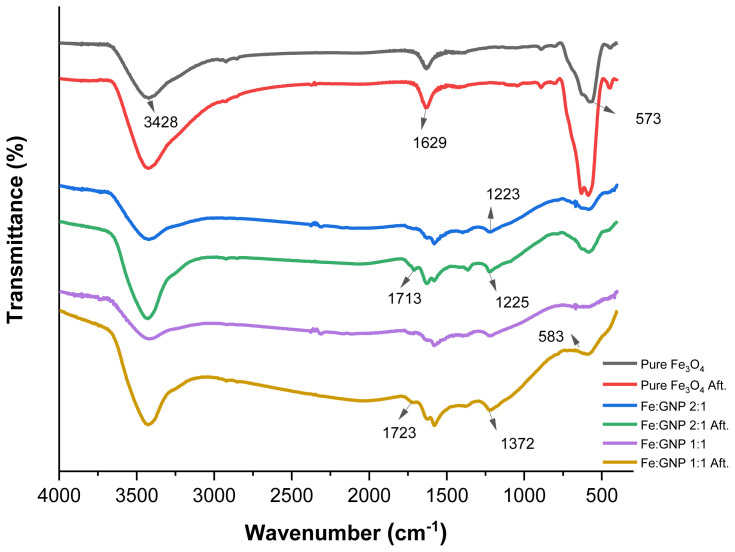
FTIR spectra of pure Fe_3_O_4_, Fe:GNP (2:1), and Fe:GNP (1:1) before and after adsorption.

The FTIR spectra of pure Fe_3_O_4_, Fe:GNP (2:1), and Fe:GNP (1:1) nanocomposites before and after adsorption are shown in Figure 1. The surface chemistries of pure Fe_3_O_4_, Fe:GNP (2:1), and Fe:GNP (1:1) were analyzed by FTIR. The spectra obtained before and after Cr(VI) adsorption are plotted in Figure 1. The presence of the peak between 3424 and 3430 cm^−1^ for all adsorbents indicates the O-H stretching vibration, and the peak at 1629 cm^−1^ indicates the aromatic skeleton C=C stretching vibration. The presence of the peak between 573 and 583 cm^−1^, which is more dominant in pure Fe_3_O_4_, can be attributed to Fe-O. This suggests a successful coupling between the Fe_3_O_4_ nanoparticles and GNPs [54]. In addition, the Fe-O density is indicative of higher iron loading. The peaks found at 1713–1729 cm^−1^ for all adsorbents other than the GNP-free adsorbent can be attributed to the stretching band of C=O in the carboxylic acid or carbonyl moieties. The appearance of two bands between 1372 and 1388 cm^−1^ and 1223 and 1225 cm^−1^ indicates that there is an interaction between the carbonyl and hydroxyl groups of the GNPs on the surfaces of the magnetic particles and that the iron oxide nanoparticles bind to the GNPs [75]. The change in the intensity of the FTIR bands is attributed to the presence of Cr(VI) adsorbed with functional groups present in the adsorbents [76]. The increase in the O-H stretching peaks after adsorption indicates adsorbed water molecules [77].

**Figure 2:**
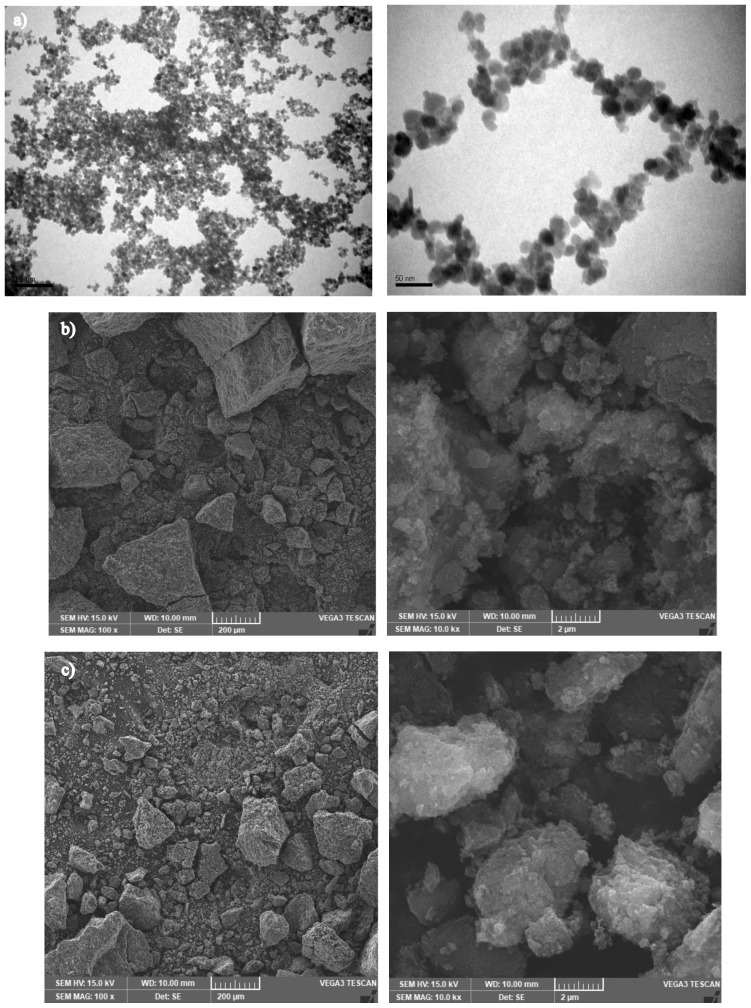
SEM images of (a) pure Fe_3_O_4_, (b) Fe:GNP (2:1), and (c) Fe:GNP (1:1).

Figure 2 shows a TEM image of pure Fe_3_O_4_ and SEM images of Fe:GNP (2:1) and Fe:GNP (1:1) nanocomposites. From the image of Fe_3_O_4_ seen in Figure 2, it was observed that the nanomaterials were aggregated and bonded with each other owing to the magnetic dipole interaction. Additionally, the prepared Fe_3_O_4_ is in the form of a smooth sphere in appearance and is estimated to have a diameter of approximately 20 nm. According to other SEM images, the Fe_3_O_4_ nanoparticles showed a homogeneous distribution together with the GNPs [48,78,79]. These SEM images reveal that the Fe_3_O_4_ and GNP successfully formed a nanocomposite structure with each other. According to the BET analysis results, the surface areas for pure Fe_3_O_4_, Fe:GNP (2:1), and Fe:GNP (1:1) nanomaterials were determined as 100.82 m^2^/g, 397.53 m^2^/g, and 437.22 m^2^/g, respectively. Thus, with the increase of the GNP ratio in the nanocomposite content, the surface areas of the materials also increased.

**Figure 3:**
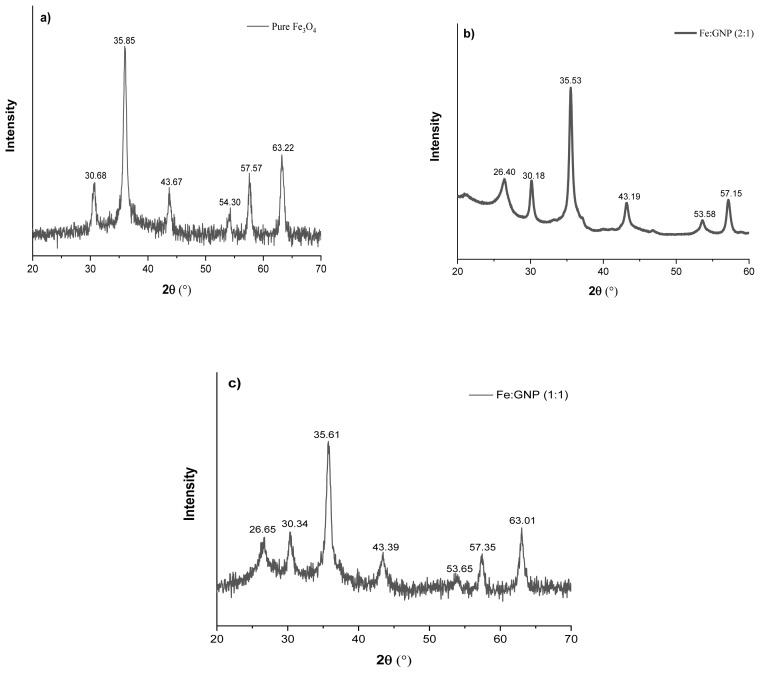
XRD patterns of (a) pure Fe_3_O_4_, (b) Fe:GNP (2:1), and (c) Fe:GNP (1:1).

The XRD patterns of pure Fe_3_O_4_, Fe:GNP (2:1), and Fe:GNP (1:1) nanocomposites are shown in Figure 3. The Fe_3_O_4_ diffraction peaks of the pure Fe_3_O_4_ adsorbent corresponded to the peaks found in the GNP-based adsorbents (Fe:GNP (2:1) and Fe:GNP (1:1)), indicating the presence of magnetite Fe_3_O_4_ [78]. The XRD patterns of adsorbents Fe:GNP (2:1) and Fe:GNP (1:1) show the characteristic peaks of the magnetite Fe_3_O_4_ and GNPs. According to the analysis results, similar characteristic peaks were observed for each nanomaterial. Specific peaks for nanocomposites were observed at 30.18°–30.68°, 35.53°–35.85°, 43.19°–43.67°, 53.58°–54.30°, 57.15°–57.57°, and 61.01°–63.22° and these results were consistent with the literature [79,80]. Additionally, specific GNP peaks were observed at 26.40° and 26.65° for the Fe:GNP (2:1) and Fe:GNP (1:1) nanocomposites [48,81]. These results confirm the presence of Fe_3_O_4_ and GNPs in the nanocomposite materials, which is in agreement with the XRD analysis results.

**Figure 4:**
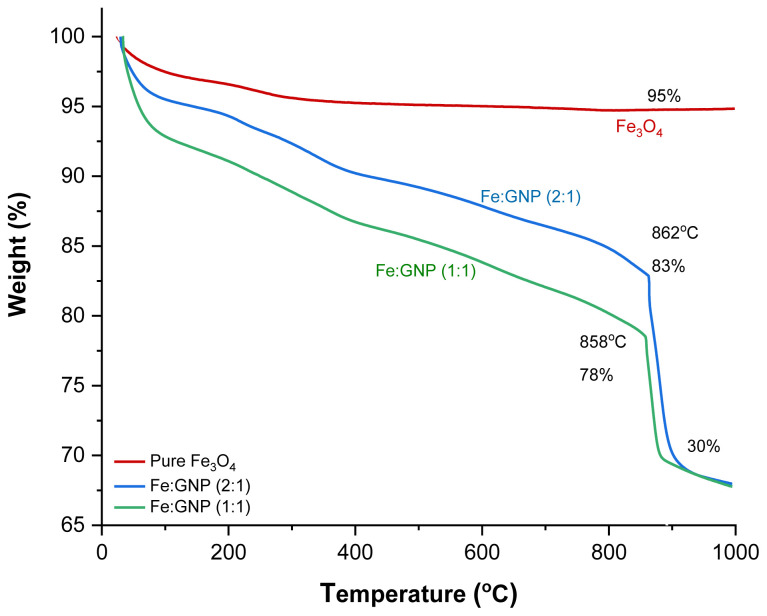
TGA plots of pure Fe_3_O_4_, Fe:GNP (2:1), and Fe:GNP (1:1).

The TGA curves of the nanocomposites as a function of temperature are shown in Figure 4. The TGA curve for Fe_3_O_4_ shows that the weight loss is about 5% in the temperature range from 25 °C to 1000 °C. The reason for this may be the amount of physically adsorbed water that pure Fe_3_O_4_ contains [82,83]. The TGA results for Fe:GNP (2:1) and Fe:GNP (2:1) show that the presence of higher amounts of Fe_3_O_4_ increases the thermal stability of the nanocomposites. Specifically, a 10% weight loss between these two materials increased the temperature by about 170 °C. The T_0.05_, T_0.10_, and T_0.20_ decomposition temperatures corresponding to 5%, 10%, and 20% weight losses and the maximum decomposition temperature (T_max_) are presented in Table 1. Based on the final thermal decomposition temperatures, it is seen from the data obtained at approximately 850 °C that the remaining material contents for Fe:GNP (2:1) and Fe:GNP (1:1) are 30% (w/w) of the total weight. These residue contents indicate Fe_3_O_4_ in the contents of the nanocomposites.

### 3.2. Adsorption process variables

**Figure 5:**
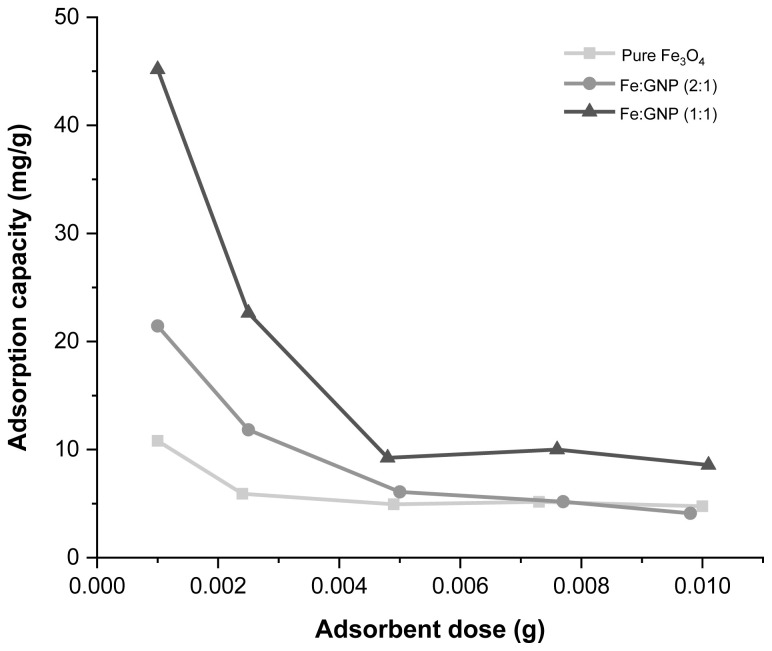
Effect of adsorbent dose of pure Fe_3_O_4_, Fe:GNP (2:1), and Fe:GNP (1:1) on Cr(VI) adsorption capacity.

Many physicochemical parameters are effective on the adsorbate/adsorbent interaction during the adsorption process and optimum conditions are determined according to those parameters. The effects of different factors such as adsorbent dose, adsorption time, solution pH, and presence of different ions on adsorption capacity were investigated for Cr(VI) removal. To determine the optimum dose of the adsorbent, different amounts of adsorbent (1, 2.5, 5, 7.5, and 10 mg) were used for adsorption with Cr(VI) solution. In Figure 5, the adsorption capacity values obtained in experiments with different amounts of magnetic adsorbents are given graphically. According to the calculated adsorption capacity values, as the amount of adsorbent decreased, the amount of Cr(VI) adsorbed per gram of adsorbent increased. In other words, as the amount of the adsorbent increased, the adsorption capacity for Cr(VI) uptake decreased. This decrease was due to the fact that active adsorbent sites are less accessible as a result of aggregated and overlapping adsorbent particles [84]. The highest adsorption capacity values were obtained when 1 mg of adsorbent and 10 mL of Cr(VI) solution were used, and the calculated values were 10.80 mg/g for pure Fe_3_O_4_, 21.43 mg/g for Fe:GNP (2:1), and 45.18 mg/g for Fe:GNP (1:1). Based on these data, the optimum adsorbent dose was chosen as 1 mg for each nanomaterial and the highest adsorption capacity was achieved with the Fe:GNP (1:1) adsorbent.

#### 3.2.1. Effect of contact time

**Figure 6:**
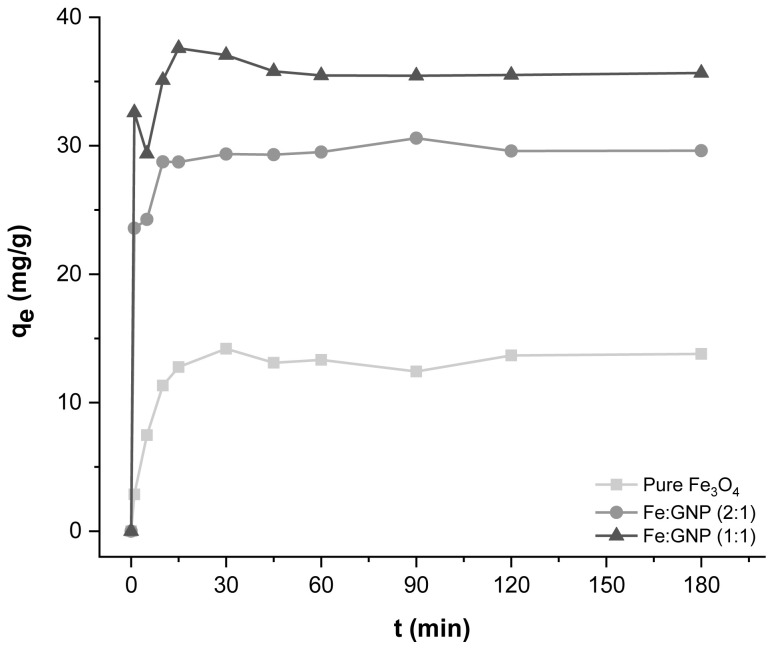
Effect of contact time on Cr(VI) adsorption capacity.

Another parameter that affects the adsorption process is contact time. To determine the optimum contact time, 1 mg of magnetic adsorbents and 10 mL of Cr(VI) solution were used for adsorption at certain time intervals (1–180 min). In Figure 6, the adsorption capacity values obtained at different time intervals in experiments with magnetic adsorbents are given. Rapid adsorption was observed in all three adsorption systems in the first stage, which can be attributed to the easily reachable active sites on the surfaces and near the edges of these nanocomposites [18]. Equilibrium was reached in approximately 90 min for all three systems and there was no significant change in q_e_ values after this point. For this reason, 90 min of contact time was sufficient for the Cr(VI) adsorption systems. Adsorption capacities for pure Fe_3_O_4_, Fe:GNP (2:1), Fe:GNP (1:1) adsorbents in 90 min were obtained as 12.43 mg/g, 30.59 mg/g, and 35.45 mg/g, respectively.

#### 3.2.2. Effect of solution pH

**Figure 7:**
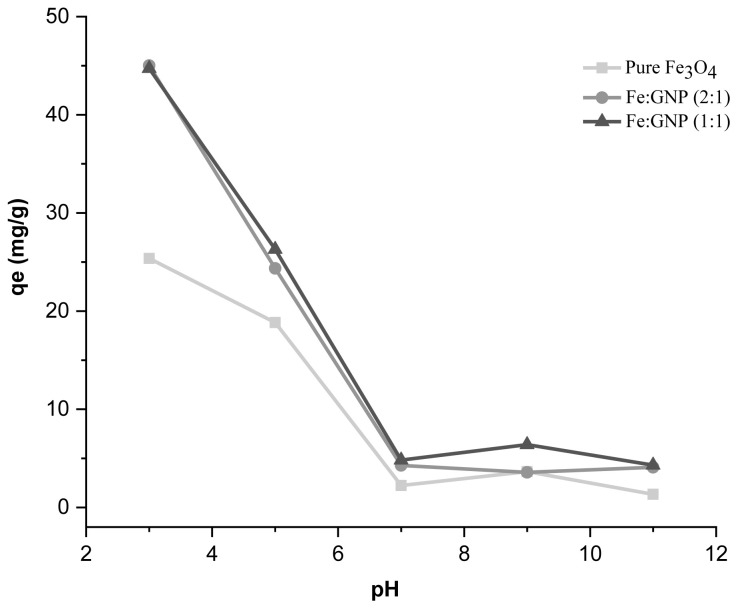
Effect of pH on Cr(VI) adsorption capacity.

The Cr(VI) solution being prepared at different pH values also affects the adsorption process. The pH of the Cr(VI) solutions was adjusted to different values (3, 5, 7, 9, and 11) using 0.1 M HCl and 0.1 M NaOH solutions. Figure 7 shows the adsorption capacity values obtained with different pH solutions. In line with these results, the amount of Cr(VI) solution per gram of adsorbent decreased as the pH value increased. Therefore, the adsorption capacity values obtained using acidic solutions at low pH values were higher than the values obtained with basic solutions. There are many studies on Cr(VI) adsorption in the literature showing that Cr(VI) adsorption behavior highly depends on the surface structure of the nanomaterials [85]. Adsorption is generally governed by electrostatic repulsion and attraction between adsorbent and adsorbate. Common chemical species of Cr(VI) in aqueous media are negatively charged (CrO_4_^2−^, Cr_2_O_7_^2−^, HCrO_4_^−^) [86]. Previous studies revealed that the acidic pH range is more suitable for the adsorption of Cr(VI). Low pH leads to an increase of H^+^ ions and results in a strong electrostatic attraction between adsorbent and chromate ions [87]. According to similar studies, the magnetic adsorbent surface is positively charged [48]. Under experimental conditions, the positive charge of adsorbents and therefore their anionic forces such as that of chromate and their attractive electrostatic interactions contributed to the adsorption capacity. Furthermore, according to the literature, it has been noted in studies using carbonaceous surface and metal oxide adsorbent materials that as the solution pH value increases, the uptake of Cr(VI) decreases [85,88]. The results obtained from these previous studies in the literature on the effect of pH on Cr(VI) uptake confirm our experimental results. The pH value of the Cr(VI) solution was approximately 5.83 (T = 16.5 °C) and no additional pH adjustment was required since high adsorption capacities were achieved in this pH range (at pH 5: pure Fe_3_O_4_, 18.83 mg/g; Fe:GNP (2:1), 24.35 mg/g; Fe:GNP (1:1), 26.30 mg/g).

#### 3.2.3. Effect of coexisting ion (NaCl)

**Figure 8:**
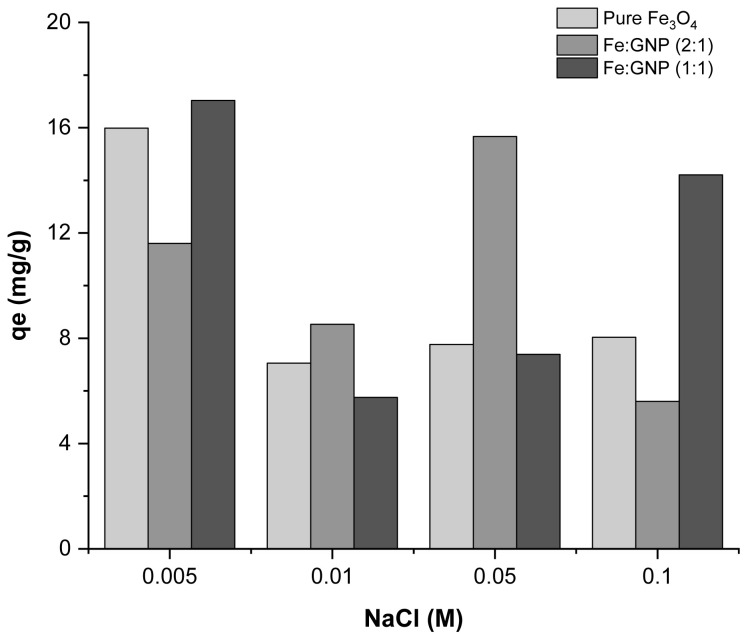
Effect of NaCl on Cr(VI) adsorption capacity.

Another parameter affecting the adsorbate/adsorbent interaction during the adsorption process is the existence of different ions in the solution. The concentrations of the solutions prepared with foreign ion-NaCl were 0.005, 0.01, 0.05, and 0.1 M. Figure 8 shows the adsorption capacity values obtained at different NaCl concentrations. NaCl ions did not cause significant differences in adsorption capacity and the adsorbents were able to perform Cr(VI) adsorption in the presence of different ions. In addition, the prepared adsorbents may have heterogeneous surfaces. Therefore, in some cases, adsorption capacity values may not be obtained stably. In general terms, while the NaCl concentration values changed from 0.005 M to 0.1 M, the Cr(VI) uptake also decreased slightly (NaCl 0.005–0.1 M: pure Fe_3_O_4_, 15.98–8.04 mg/g; Fe:GNP (2:1), 11.61–5.60 mg/g; Fe:GNP (1:1): 17.04–14.21 mg/g). According to similar studies in the literature, Cr(VI) removal decreased with the addition of different metal ions to the solution. This reduction suggests that it may be due to the competition of metal ions for surface binding sites [87].

### 3.3. Kinetic model studies

Adsorption kinetic studies are important for providing information about the adsorption mechanism of Cr(VI) on magnetic nanocomposites. Optimization of the adsorption system requires a detailed understanding of the driving forces governing the mutual effects between the adsorbate and adsorbent [87].

**Figure 9:**
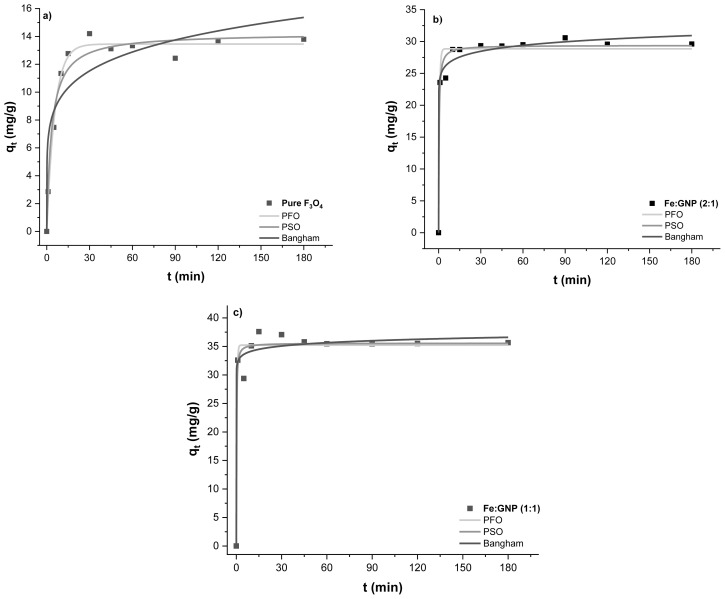
Kinetic models of (a) pure Fe_3_O_4_, (b) Fe:GNP (2:1), and (c) Fe:GNP (1:1).

Equilibrium times of the adsorption systems were determined at room temperature (25 °C) and the adsorption kinetics of the adsorbents on Cr(VI) were investigated. The graphs obtained by applying the nonlinear PFO, PSO, and Bangham kinetic models [83] to the experimental data with Eq. (2), Eq. (3), and Eq. (4), respectively, are shown in Figure 9. Here, q_t_ (mg/g) is the adsorption capacity at time t, the rate constant values are k_1_ (min^−1^) and k_2_ (g/mg min) for the PFO and PSO kinetic models, k is the Bangham constant, υ is the Bangham parameter, and t (min) is the contact time. The parameters of the models calculated using these equations are presented in Table 2.


(2) $${\text{q}}_{\text{t}}={\text{q}}_{\text{e}}.(1-{\text{e}}^{-{\text{k}}_{1}\text{.t}})$$

(3) $${\text{q}}_{\text{t}}=\frac{{\text{q}}_{\text{e}}^{2}{\text{.k}}_{2}\text{.t}}{1+{\text{k}}_{2}{\text{.q}}_{\text{e}}\text{.t}}$$

(4) $${\text{q}}_{\text{t}}=\text{k}{\text{.t}}^{\upsilon }$$

The PFO, PSO, and Bangham kinetic models were used to examine the adsorption mechanism and the PSO was the most suitable model for the experimental data with a relatively higher regression coefficient for each adsorption system (R^2^ > 0.97). The PSO kinetic model supposes that the adsorption system is chemisorptive in nature. In other words, this model shows that the rate-limiting step can be chemisorption with the inclusion of valence changes through electron sharing or exchange between the adsorbent and adsorbate [18,87]. The experimental data for adsorption capacity and the model parameters were found to be compatible with each other (pure Fe_3_O_4_: q_t_ = 12.43 mg/g, q_e_ = 13.45 mg/g; Fe:GNP (2:1): q_t_ = 30.58 mg/g, q_e_ = 28.86 mg/g; Fe:GNP (1:1): q_t_ = 35.45 mg/g, q_e_ = 35.56 mg/g). In addition, Table 2 shows that the Cr(VI) uptake rate decreases in the following order: Fe:GNP (1:1) > Fe:GNP (2:1) > pure Fe_3_O_4_. The third model examined was the Bangham kinetic model, which is a generalization of the Weber–Morris model and explains the slow diffusion step of the adsorption process in the pores of adsorbent materials [83,89]. The Bangham model, with high regression coefficients, especially for the adsorption of Cr(VI) on the Fe:GNP (2:1) and Fe:GNP (1:1) adsorbents (R^2^ ≥ 0.97), shows that the model is compatible with the experimental kinetic data and that the adsorption takes place by diffusion into the pores in the adsorbents [83,90].

### 3.4. Equilibrium models

**Figure 10:**
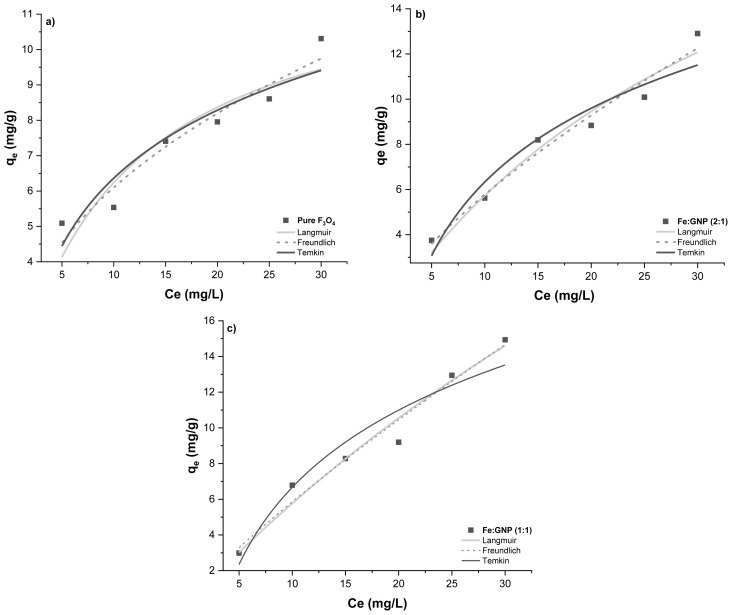
Isotherm models of (a) pure Fe_3_O_4_, (b) Fe:GNP (2:1), and (c) Fe:GNP (1:1).

Adsorption isotherms were obtained to examine the mechanisms of the adsorption systems at room temperature (25 °C). The graphs obtained by applying the nonlinear Langmuir, Freundlich, and Temkin isotherm models to the experimental data with Eq. (5), Eq. (6), and Eq. (7), respectively, are shown in Figure 10. Here, K_L_ (L/mg) is the Langmuir isotherm constant, K_F_ is the (mg/g)(L/mg)^1/n^ Freundlich isotherm constant and 1/n is the heterogeneity factor showing the adsorption density, A_T_ is the Temkin isotherm equilibrium binding constant, b_T_ is the Temkin isotherm constant, R (8.314 J/mol K) is the gas constant, and T (K) is temperature. The isotherm model parameters calculated using these equations are presented in Table 3.


(5) $${\text{q}}_{\text{e}}=\frac{{\text{K}}_{\text{L}}\;{\text{q}}_{\text{m}}\;{\text{C}}_{\text{e}}}{1+{\text{K}}_{\text{L}}\;{\text{C}}_{\text{e}}}$$

(6) $${\text{q}}_{\text{e}}={\text{K}}_{\text{F}}\;{\text{C}}_{\text{e}}^{1/\text{n}}$$

(7) $${\text{q}}_{\text{e}}=\frac{\text{R}\times \text{T}}{{\text{b}}_{\text{T}}}\times \text{ln}({\text{A}}_{\text{T}}\times {\text{C}}_{\text{e}})$$

The Langmuir isotherm model explains that the thickness of the adsorbed layer is the same and equivalent to the adsorption process; in other words, monolayer adsorption occurs. It also assumes that the adsorption is homogeneous since each molecule has constant enthalpy and activation energy [91]. The maximum adsorption capacities of the pure Fe_3_O_4_, Fe:GNP (2:1), and Fe:GNP (1:1) adsorbents determined by the Langmuir isotherm model were 12.71, 27.03, and 62.27 mg/g, respectively. According to the regression coefficients, the best-fitting model for all adsorption systems is the Freundlich isotherm model (R^2^ ≥ 0.94). It is known that if the 1/n value is less than 1, the adsorption process yields a positive result. The 1/n values for all three adsorption systems ranged from 0 to 1, indicating that Cr(VI) adsorption can be performed properly with magnetic adsorbents. The Freundlich isotherm model explains that it is possible to apply multilayer adsorption without being limited to monolayer formation and that the heterogeneous surface has energy-containing regions [91,92]. The isotherm parameters show that the magnetic adsorbents adsorb Cr(VI) molecules in different energy regions as multilayers. Another model that indirectly describes adsorbent/adsorbate interactions is the Temkin isotherm. According to this model, the adsorption of the Cr(VI) adsorbate with magnetic adsorbents is based on a chemical absorption process [83,93]. Calculated using the Temkin isotherm model, b_T_ (pure Fe_3_O_4_, Fe:GNP (2:1), and Fe:GNP (1:1): 893.6, 525.8, and 397.1 J/mg) is related to the heat of sorption, which indicates the physicochemical nature of the system [94]. In this equilibrium study, the effect of the initial concentration of the Cr(VI) solution was also observed. As the concentration of the Cr(VI) solution increased, the adsorption capacity also increased due to the adsorbent/adsorbate interaction.

A summary of many studies on Cr(VI) adsorption with magnetic adsorbents in the literature is presented in Table 4. Compared with the results of these previous studies, higher adsorption capacity values were obtained in this study, especially for the GNP-based nanocomposites. It was determined that the maximum adsorption capacity increased as the amount of GNPs in the magnetic nanocomposites increased.

## 4. Conclusion

Magnetic adsorbents were prepared and used successfully for the removal of toxic Cr(VI) from aqueous solutions. Fe:GNP nanocomposites compatible with the literature were prepared and characterized using FTIR, XRD, SEM, BET, and TGA techniques. The effect of the existence of GNPs on the adsorption systems was examined by changing the Fe:GNP ratio of the magnetic materials. As the GNP ratio in the nanomaterial content increased, the adsorption process was positively affected and the adsorption capacities increased for all processes. BET analyses also supported these results, as higher adsorption efficiencies were achieved with increased surface areas. Various parameters affecting the adsorbate/adsorbent interaction during the adsorption process, including adsorbent dose, contact time, initial Cr(VI) solution concentration, pH, and the existence of coexisting (NaCl) ions, were investigated. For all adsorption processes, the optimal adsorbent dose was 0.1 g/L and the adsorption equilibrium time was 90 min. Adsorption kinetics were investigated using the PFO, PSO, and Bangham kinetic models and the PSO kinetic model was the most compatible model according to both experimental results and correlation coefficients (R^2^ ≥ 0.97). The Langmuir, Freundlich, and Temkin isotherm models were used to examine the adsorption mechanism and the Freundlich isotherm was the most suitable model for the experimental data with a relatively higher correlation coefficient (R^2^ ≥ 0.94). The maximum adsorption capacities for pure Fe_3_O_4_ (Fe:GNP 1:0), Fe:GNP (2:1), and Fe:GNP (1:1), respectively, were obtained as follows: 12.71 mg/g, 27.03 mg/g, and 62.27 mg/g. The pH effect depending on the adsorbent/adsorbate surface structure was investigated and it was concluded that solutions with low pH values were more suitable for adsorbent-Cr(VI) systems. As a result of this study, magnetically modified GNP materials can be considered as potential favorable adsorbents for the removal of various pollutants such as Cr(VI) from polluted water.

## Figures and Tables

**Table 1:** Decomposition temperatures at weight losses obtained from TGA curves of nanocomposites.

Sample	T_0.05_ (°C)	T_0.10_ (°C)	T_0.20_ (°C)	T_max_ (°C)
Pure Fe_3_O_4_	402.9	-	-	-
Fe:GNP (2:1)	130.2	422.9	864.34	902.9
Fe:GNP (1:1)	59.1	251.0	805.1	882.1

**Table 2:** PFO, PSO, and Bangham kinetic model parameters of nanocomposite-Cr(VI) adsorption systems.

Cr(VI) adsorption	Pure Fe_3_O_4_	Fe:GNP (2:1)	Fe:GNP (1:1)
PFO			
q_e_ (mg/g)	14.23	28.86	35.22
k_1_ (min^−1^)	0.180	1.692	2.593
R^2^	0.97	0.96	0.96
PSO			
q_e_ (mg/g)	13.45	29.40	35.56
k_2_ (g/mg min)	0.021	0.113	0.223
R^2^	0.98	0.98	0.97
Bangham			
k	6.67	24.32	32.35
υ	0.16	0.05	0.02
R^2^	0.84	0.98	0.97

**Table 3:** Langmuir, Freundlich, and Temkin isotherm model parameters of nanocomposite-Cr(VI) adsorption systems.

Cr(VI) adsorption	Pure Fe_3_O_4_	Fe:GNP (2:1)	Fe:GNP (1:1)
Langmuir			
q_m_	12.71	27.03	62.27
K_L_	0.096	0.027	0.010
R^2^	0.87	0.96	0.97
Freundlich			
K_F_	2.29	1.19	0.86
n	2.35	1.46	1.20
R^2^	0.94	0.97	0.97
Temkin			
A_T_	0.99	0.38	0.29
b_T_	893.6	525.8	397.1
R^2^	0.89	0.93	0.93

**Table 4:** Summary of studies in the literature on Cr(VI) removal with magnetic adsorbents.

Adsorbents/composite materials	Maximum adsorption capacity, q_m_	Reference
Magnetite–polyethyleneimine–montmorillonite	8.8 mg/g	[64]
N-doped porous carbon with magnetic nanoparticles	16 mg/g	[56]
Mesoporous magnetic carbon nanocomposite	3.74 mg/g	[61]
Magnetic magnetite (Fe_3_O_4_)	34.87 mg/g	[63]
Amorphous FeB alloy-modified magnetite nanocomposites	38.9 mg/g	[69]
Fibrillar magnetic carbon	43.17 mg/g	[60]
Particulate magnetic carbon	15.88 mg/g	
Chicken eggshell-coated magnetic adsorbent	38.76 mg/g	[66]
Magnesium ferrites MgFe_2_O_4_	10 mg/g	[73]
Magnesium-zinc ferrites Mg_0.8_Zn_0.2_Fe_2_O_4_	19 mg/g	
Magnesium-zinc ferrites Mg_0.6_Zn_0.4_Fe_2_O_4_	25 mg/g	
Magnesium-zinc ferrites Mg_0.4_Zn_0.6_Fe_2_O_4_	26 mg/g	
Magnesium-zinc ferrites Mg_0.2_Zn_0.8_Fe_2_O_4_	34 mg/g	
Zinc ferrites ZnFe_2_O_4_	10 mg/g	
Magnetic activated carbon from animal bone waste	27.86 mg/g	[70]
Pure Fe_3_O_4_	12.71 mg/g	This study
Fe:GNP (2:1)	27.03 mg/g	This study
Fe:GNP (1:1)	62.27 mg/g	This study
